# Informing equitable noncommunicable disease prevention policies through lived experience: a scoping review of research approaches

**DOI:** 10.1186/s12961-025-01348-2

**Published:** 2025-11-27

**Authors:** Christina Zorbas, Jacqueline Monaghan, Jennifer Browne, Phoebe Nagorcka-Smith, Andrew D. Brown, Dheepa Jeyapalan, Steven Allender, Anna Peeters, Rebecca Christidis, Kathryn Backholer

**Affiliations:** 1 Global Centre for Preventive Health and Nutrition, Institute for Health Transformation, Deakin University, 1 Gheringhap Street, Geelong, VIC 3220 Australia; 2 Good Shepherd Australia New Zealand, Melbourne, VIC Australia; 3 The Victorian Health Promotion Foundation (VicHealth), Melbourne, VIC Australia

**Keywords:** Lived experience, Health equity, Health policy, NCD prevention, Scoping review

## Abstract

**Background:**

People experiencing marginalisation tend to be systematically excluded from policy decisions. Engaging people with lived experiences of marginalisation is increasingly considered critical for developing equitable and effective noncommunicable disease (NCD) policies. It remains unclear how the voices and experiences of people who are harmed by systems of marginalisation due to gender, ethnicity, sexuality, disability and social position have been included in NCD prevention policies.

**Methods:**

We conducted a systematic scoping review, grounded in constructivist epistemology and critical theory. Five overarching search terms were applied across Medline, Academic Search Complete, CINAHL, and Global Health to describe priority populations, lived experience, participatory research, NCDs and policy. Articles were included if they involved research engaging the lived experiences of local communities and people experiencing marginalisation in high-income countries to inform equitable NCD prevention policies. Factors affecting the inclusion of lived experience were meta-analysed thematically across included studies.

**Results:**

In total, 49 articles met the eligibility criteria – focused on NCD prevention related to nutrition (35% of studies), NCDs in general (20%), physical activity (12%), tobacco (10%), obesity (10%), mental health (8%) and alcohol (4%). The majority (67%) of research was conducted in the United States, followed by Canada (14%), Australia (6%), Europe (8%), and the United Kingdom (4%). Study participants included Black, Hispanic, and other multicultural communities (52% of studies), people in regional or rural areas (37%), First Nations peoples (22%), residents in low-income areas (28%), people receiving a low income (20%), women only (9%) and people experiencing disability (2%). Studies typically involved policy advocacy to local governments (79%), often supported by local coalitions (22%). Factors underpinning inclusive NCD prevention policymaking included having a strong purpose for engaging with lived experiences of marginalisation, fostering a deep understanding of culturally safe practices, addressing institutional tensions and power imbalances, and co-creating mechanisms for impact (e.g. policy networks and safe spaces).

**Conclusions:**

Best practice approaches for including people with lived experiences of marginalisation in NCD prevention policies and research are lacking and should continue to be developed. National-level leadership, genuinely supporting communities, and being aware of one’s own role in social change are necessary to improve institutional practices that systemically exclude diverse experiences.

**Supplementary Information:**

The online version contains supplementary material available at 10.1186/s12961-025-01348-2.

## Background

### Inequalities in the distribution of noncommunicable diseases (NCDs)

Social factors such as education, income, geography, ethnicity, gender and sexuality are associated with inequalities in the distribution of noncommunicable diseases (NCDs). NCDs include cardiovascular diseases, type 2 diabetes and cancers, which are largely preventable owing to modifiable dietary risks, a high body mass index, physical inactivity, and alcohol and tobacco use [1, 2]. Despite their rich diversity and strengths, people who are most harmed by systems of marginalisation tend to face the highest prevalence of many NCDs and their risk factors [3–6]. Systems of marginalisation are defined as the structures, policies and practices that systematically exclude and discriminate against certain populations to create persistent social and health inequalities [7].

A systematic review of 47 studies in Organisation for Economic Co-operation and Development (OECD) countries found that a low socioeconomic position, defined using various measures such as education, income, and occupation, was consistently associated with NCD-related morbidity and mortality [8]. In addition, a 2024 global systematic review indicated that First Nations populations had double the risk of experiencing multiple chronic disease-related morbidities compared with non-First Nations populations [9]. African Americans have similar lived experiences. For example, in 2023, African American adolescents had a 50% higher likelihood of experiencing obesity than white adolescents, with female adolescents being twice as likely as their white counterparts [10]. Women have also been found to experience higher cardiovascular morbidity than men after acute events [11], especially women who are non-white, have low socioeconomic positions and are geographically marginalised [12].

Herein, we use the terminology people experiencing marginalisation to collectively describe diverse social groups facing structural barriers to NCD-related health. However, analyses must not be deficit-focused and should recognise the expertise, strengths and resilience of these communities, including First Nations peoples and multicultural communities, in directing policy to improve health and reduce inequities. To support all communities to experience good health and wellbeing, public policy and decision-making should prioritise actions to support community self-determination and reduce the underlying imbalances in power, money, resource distribution and involvement in decision-making [1, 13].

### Participatory NCD policymaking

Policy refers to the process of developing plans of action to address an issue, often involving policy advocacy, agenda setting, policy formulation, adoption, implementation and evaluation [14]. Scholarship on public policy suggests that public representation and participation in policymaking, especially among people who experience marginalisation, are necessary to design and deliver policy solutions that are fit-for-purpose and can achieve equitable outcomes [15]. For instance, the recent *Australian National Preventive Health Strategy 2021–2030 *[16] indicates that for population groups experiencing a disproportionate burden of disease:

Shared decision-making, strategic partnerships and involving people with lived experience at the heart of policy development and implementation are key to creating meaningful change (National Preventive Health Strategy 2021–2023, Commonwealth of Australia, p. 21).

Participatory policymaking supports the fulfilment of human rights and self-determination [17], contributing to systems change through shifts in power dynamics, resource flows and mental models [18]. Benefits of participatory policymaking for institutions include increasing government access to on-the-ground knowledge to identify effective solutions and building community capacity to sustainably lead changes through partnerships [15]. Some examples of participatory policymaking in relation to NCD prevention, where community voice informs policy advocacy and decisions, exist among predominantly African American and Hispanic communities in the United States of America (USA) and among First Nations communities in Australia [19, 20]. There is also an increasing focus in literature on including the voices and lived experiences of young people in NCD prevention [21]. For the purposes of this research, we define lived experience as the first-hand knowledge and perspectives of people experiencing marginalisation.

Systematic reviews suggest that whilst community engagement in NCD prevention has been explored by researchers and policymakers [22–24], the extent to which policymaking can be influenced by people with lived experience of marginalisation is unclear. According to Arnstein’s Ladder (1969), an influential conceptual framework describing the varying degrees of participation in government decision-making, “nonparticipation” and “tokenism” are weak but common forms of participation, resulting in little change to the status quo [25]. In contrast, “citizen power” through citizen control and partnerships in policymaking is more likely to inform the development of impactful policies that redistribute power but is less likely to be pursued by governments than weaker forms of participation [25]. Community-based participatory research (CBPR) has evolved over time to provide other examples of how to strengthen power sharing, partnerships and governance practices with a view to facilitate better community engagement and community-driven research for health equity [26]. At their core, community-based research methodologies aim to challenge the prioritisation of academic knowledge by respecting and privileging community knowledge in decision-making [26].

### Research rationale and aims

Calls to involve people who experience marginalisation in public health policy are based on indications that co-created policies are more likely to be effective than those developed by people without this lived experience [25]. Although some studies [19, 22, 23] have examined approaches to including community voices and experiences in policymaking, the breadth of this research has not been systematically synthesised to understand the state of the field regarding equitable prevention of NCDs. Moreover, whilst targeted policymaking processes increasingly seek to include communities of interest in decision-making, the extent to which population-level NCD prevention policies seek to include diverse community input is less clear, despite policy potential to widen or narrow health inequities [27]. We sought to address this gap by conducting a systematic scoping review that aims to:

1. Describe research methods that can engage people with lived experience of marginalisation to inform equitable governmental NCD prevention policymaking in high-income countries.
2. Identify factors determining meaningful and impactful conduct of lived experience research for NCD prevention policy.

## Methods

### Study design and theoretical orientation

This review used the Preferred Reporting Items for Systematic Reviews and Meta-Analyses (PRISMA) scoping review guidelines [28]. A scoping review is most suitable and useful to understand research gaps and priorities when studying emerging topics. We are guided by a constructivist epistemology and a critical theory lens in our endeavour to challenge health injustices [29], as demonstrated by positionality and reflexivity statement (Positionality and reflexivity section).

### Search strategy and screening

We searched Medline, Academic Search Complete, CINAHL, and Global Health with terms pertaining to (1) populations experiencing social inequality and discrimination due to gender, ethnicity, indigeneity, sexuality, disability and socioeconomic position; (2) lived experience; (3) citizen participation; (4) NCDs and their risk factors; and (5) policy. Supplementary Table S1 outlines the full set of search terms used for Medline, including MeSH terms. The first 200 results from Google Scholar (which are likely to produce the most relevant results [30]) and citations associated with included articles, including grey literature, were also screened to identify relevant studies. Articles retrieved from this search strategy were uploaded into Covidence [31], and one author (C.Z.) screened titles and abstracts on the basis of the eligibility criteria.

### Eligibility criteria and selection

To be included, studies needed to:

1. Involve populations with lived experience of marginalisation due to gender, ethnicity, sexuality, disability, geography, income and/or social position.
2. Provide detail on a method or approach used to engage people with lived experience in policies aiming to prevent NCDs and/or their risk factors. We were interested in any aspect of the policy process including policy advocacy, agenda setting, policy formulation, adoption, implementation and evaluation [14].
3. Describe community engagement methods that enable people to participate meaningfully or actively in research and/or policy related to the primary prevention of NCDs.
4. Report primary research, literature reviews of primary research or reports from established government or non-government organisations.
5. Explicitly focus on one or more of the different aspects of policymaking (described above) in organisational or government-led NCD prevention policy processes.
6. Be conducted in high-income OECD countries [32] where the prevalence and determinants of NCDs are similar, there are comparable populations who experience marginalisation, and socioeconomic contexts, research systems and recommendations regarding participatory policymaking are likely to be transferable.

Studies were excluded if they were not published in English, solely focused on individual behaviour change strategies, and/or were study protocols or opinion pieces. All relevant full text articles were independently screened and selected by two authors (C.Z. and J.M.), with conflicts resolved through discussion.

### Data extraction and quality appraisal

Study characteristics were recorded using a data extraction table in Microsoft Excel. One reviewer (C.Z.) extracted each study’s aim(s); country; study design; NCD topic; social indicator/s (income, area-level income, occupation, education, remoteness, First Nations, ethnicity other than First Nations, gender, sexuality, disability, other); sample size; recruitment methods; youth participation (yes/no); stage of policy cycle; and policy level (local/state/federal/organisational). Methods for collecting data on lived experience were coded according to a previous study [33]. Quality appraisal was undertaken by J.M. using the 10 Qualitative checklist items in the Critical Appraisal Skills Programme (CASP) Quality Appraisal Tool [34] – itself developed without the voices of communities. C.Z. cross-checked the quality appraisal to resolve uncertainties. All studies were included in the narrative synthesis regardless of quality to summarise lessons learned.

### Narrative synthesis

The extent and nature of how research has gathered first-hand knowledge and insights from people experiencing marginalisation to inform NCD prevention policymaking was narratively summarised. To provide a comprehensive overview of the factors that affect the inclusion of lived experience in research pertaining to NCD prevention policies, we thematically analysed the included studies. All sections of the included manuscripts were inductively coded by the lead researcher (C.Z.) using NVivo, and codes were categorised to produce higher-level major themes with sub-themes and were reviewed and agreed upon by the research team [35].

### Positionality and reflexivity

Our research team included several investigators who have experienced various forms of social inequity whilst also predominantly consisting of white Australians with academic power and privilege. The research team also recognises the limitations of using the CASP quality appraisal tool to examine the cultures of researchers, their Western knowledge and education power, and their control over the research design. This is also the case for the PRISMA scoping review guidelines. To try to mitigate these limitations, we invited two experts in cultural safety to review our work (co-author D.J. and acknowledgement for M.L.).

## Results

**Fig. 1:**
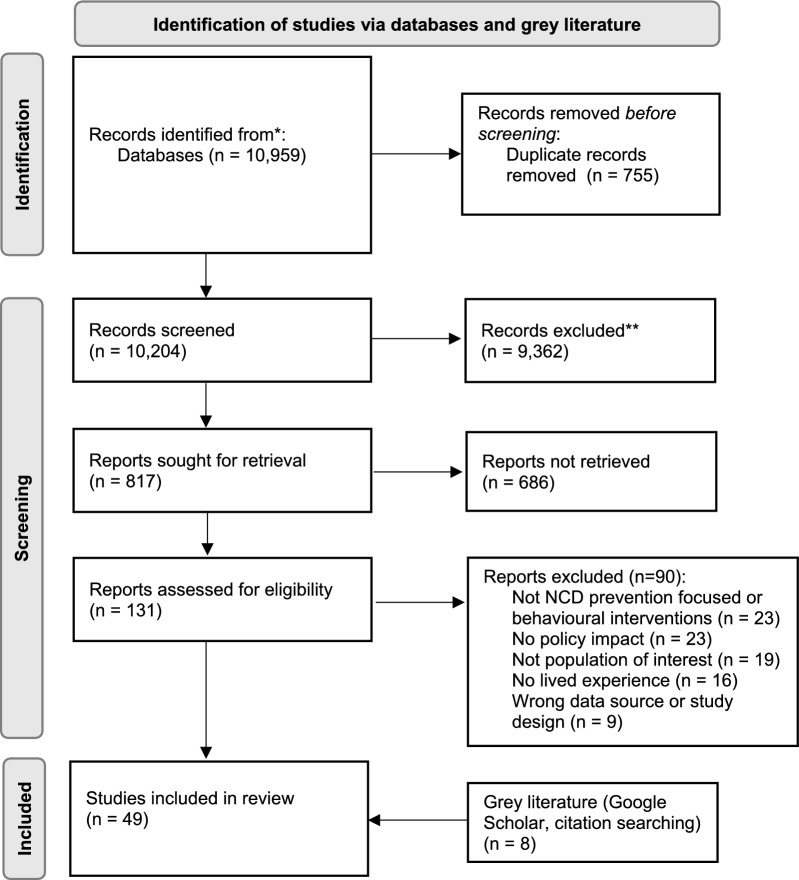
PRISMA 2020 flow diagram outlining study selection

Following the systematic screening of 10 959 search results, 49 academic articles were included in this review (Fig. 1 and Supplementary Table S2). Across the included articles, the mean quality appraisal score was 8 out of 10 CASP criteria, with 42% of studies scoring 9 or more and 9% scoring 5 or less out (Supplementary Table S3). Study quality was most reduced by inadequate or unclear description of researcher positionality (94% of studies), ethical issues (37%), recruitment strategy (31%) and data analysis (20%).

### Study characteristics

**Fig. 2:**
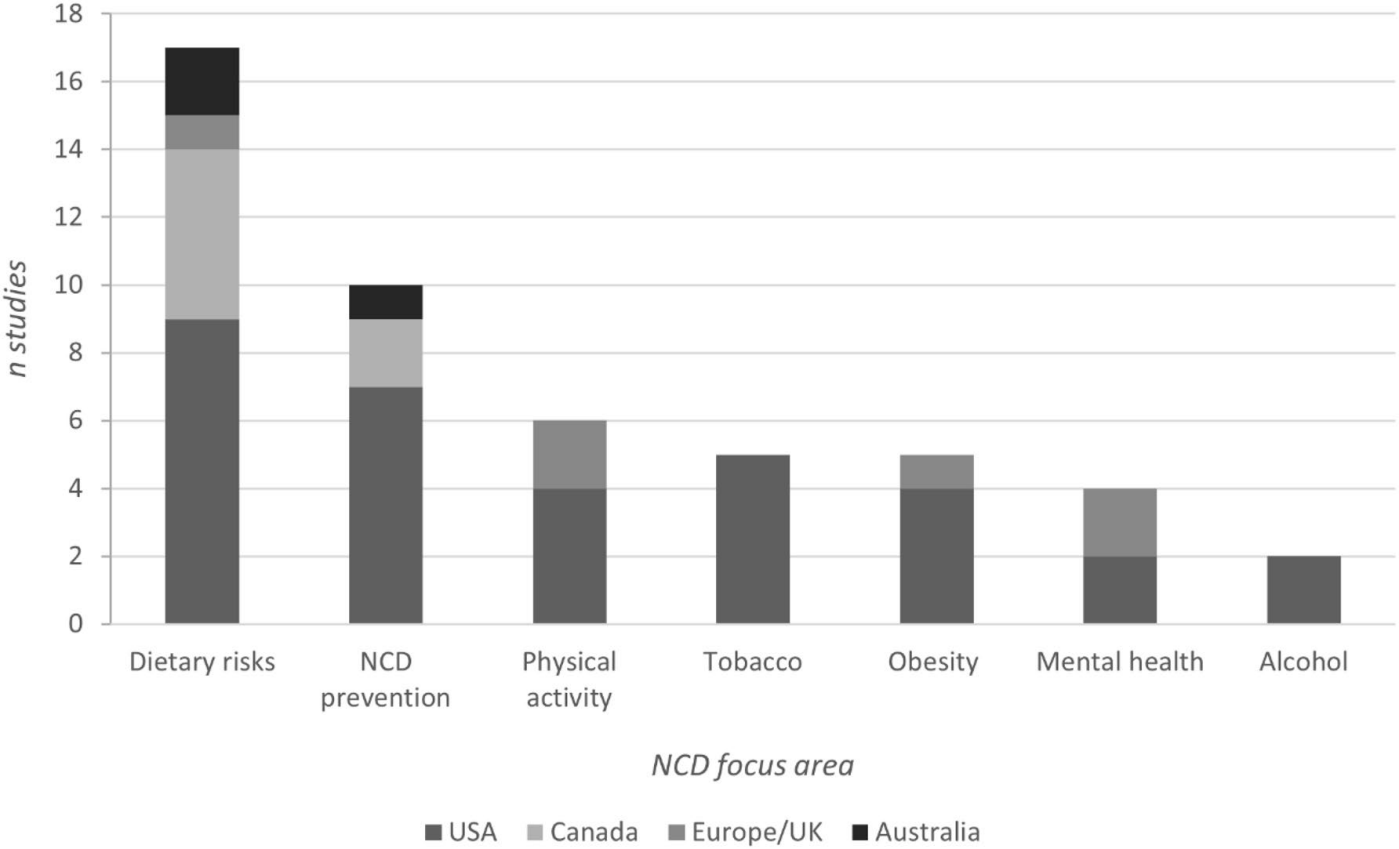
NCD prevention policy areas and countries represented in included studies

More than one third (35%) of included studies spanned the policy areas of nutrition, 20% focused on NCD prevention generally, 10% on tobacco, 10% on obesity, 12% on physical activity, 8% on mental health and 4% on alcohol (Fig. 2). Two thirds of the research was conducted in the USA, followed by Canada (14%), Australia (6%), Europe (8%), and the United Kingdom (UK; 4%), with all studies published from 2003 onwards. NCD prevention policies were more often targeted (64%) rather than population-wide policies (8%), with approximately one quarter involving both policy types. Approximately half of the study participants included Black, Hispanic, and other multicultural communities (52% of studies), followed by studies frequently including people in regional or rural areas (37%); First Nations peoples in Canada, the USA and Australia (22%); residents in low-income areas (28%); people receiving a low income (20%); women only (9%); and people experiencing disability (2%). In total, 37% of studies engaged young people.

**Fig. 3:**
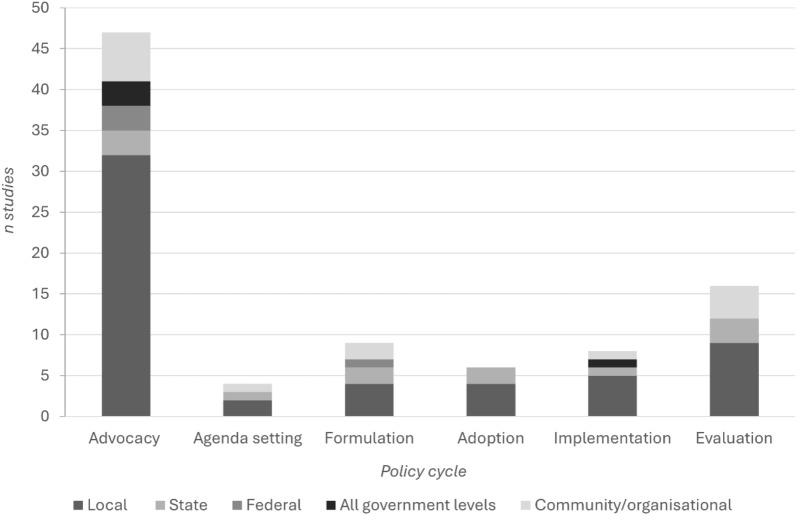
Aspects of the policy process represented in included studies

On average, studies recruited 72 participants, with sample sizes ranging from 6 to 600. Interviews (39%), photovoice (37%) and focus groups (31%) were the most commonly applied methods to collect people’s lived experience. To a lesser extent, visual and systems mapping (16%), observation (10%) and consensus panel (4%) methods were used. As Fig. 3 shows, the most common stage and level of policy described in included studies was advocacy at the local government level. While 68% of studies involved policy advocacy, 23% focused on policy evaluation, 9% on policy formulation, 9% on policy implementation, 8% on policy adoption and 6% on policy agenda setting. In addition, 22 of the included studies used coalitions to drive participation in the policy process, and 79% of studies targeted policy at the local government level, followed by 21% at community/organisational, 17% at state government, 8% at federal government and 6% at intergovernmental levels.

### Factors affecting the inclusion of lived experience in research informing NCD-prevention policy

**Fig. 4:**
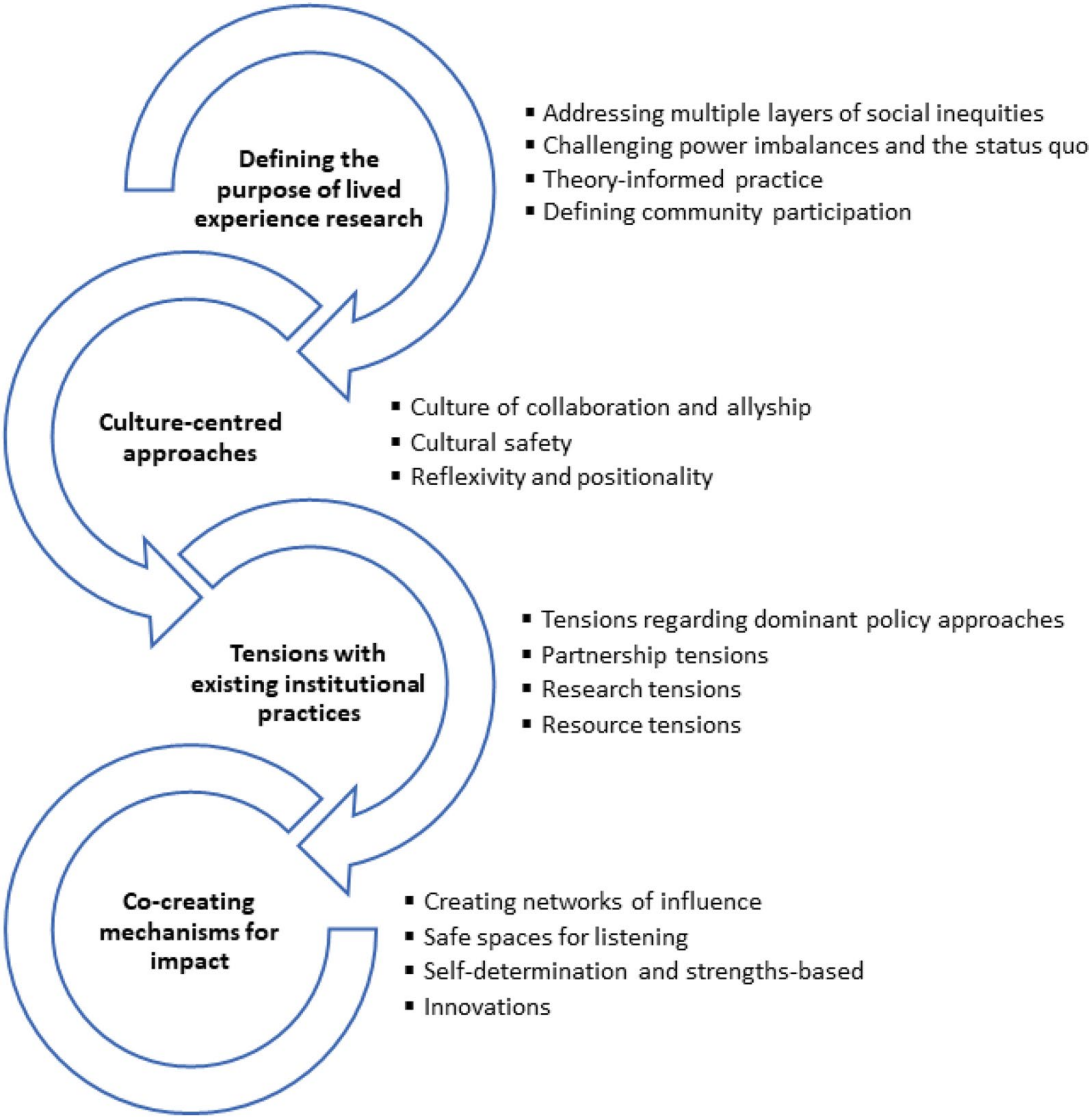
Thematic summary of factors affecting the inclusion of community lived experiences in research to inform equitable NCD prevention policies

Four major themes and 15 sub-themes (italicised within the text below) were identified through thematic analysis of included studies (Fig. 4). Major themes related to: (i) defining the purpose of the lived experience research; (ii) taking a culture-centred approach; (iii) addressing tensions with existing institutional practices; and (iv) co-creating mechanisms for impact. Below we describe each theme in detail.

#### Defining the purpose of the lived experience research

Researchers and participants described several reasons for including lived experience of marginalisation in research to inform NCD prevention policy. Almost all studies recognised the potential for this work to *shine a light on the multiple intersecting layers of social and economic inequities (i.e. the social determinants of NCDs and health; sub-theme 1).* The root causes described varied across studies but collectively included understanding the historical context of how Western dominance, colonisation and neoliberal development policies have harmed certain communities and limited diversity of representation. Such political contexts have provided grounds for opportunity deserts [36] and thus competing community priorities, including addressing employment, poverty, income inequality, inequitable land access, inadequate social supports, low literacy, social exclusion, shame and stigma, abuse and violence, limited access to services, and cost of living crises. The intersecting layers of inequities were found to perpetuate feelings of powerlessness in some studies [36–40]. As such, two studies [37, 41] indicated that community healing was a key aim of lived experience research.

*Increasing community power by challenging power imbalances in the current status quo of decision-making (sub-theme 2)* was described in studies as another reason why lived experience should be included in NCD prevention policy research. More specifically, studies indicated that community power could be increased by generating a collective community voice that valued and respected community knowledge and expertise [37, 40, 42–45], challenged knowledge authority (i.e. democratised knowledge and decision-making) [37, 40, 46, 47], contributed new strengths-based narratives [40–42, 44, 46, 48–53], supported self-determination [36–38, 40, 42, 46, 50, 54–56], fostered a sense of belonging [40, 41, 46], enabled truth telling (as opposed to silencing) [44, 45, 47, 57], and highlighted weaknesses in the effectiveness of current policies and processes [37, 41].

In defining the purpose of lived experience research in NCD prevention policy, CBPR [42, 46, 52, 54] and participatory action research (PAR) [40, 43, 44, 46, 47, 54, 56] were the most cited (49%) as guiding frameworks. *Multiple other theories and frameworks, often equity-oriented, were used to inform studies (sub-theme 3)* – including deliberative democracy theory [45], critical theory [40, 46], feminist theory [40, 46], and normalisation process theory [37, 40, 43]. Across the reviewed studies, *community participation was inconsistently defined, especially in terms of capacity building and empowerment (sub-theme 4)*. Some common ideas underpinning these concepts included enabling leadership whereby the community identifies local priorities and solutions [37, 38, 41, 44, 46–50, 52, 53, 55, 57, 58], shared decision-making [36, 37, 40–43, 45, 47, 50–54, 56, 57, 59], self-determination and agency [36–38, 40, 42, 46, 50, 54–56], and political organising and participation by communities. Working in partnership with community members and organisations was typically viewed as essential to enable regular knowledge exchange and meet people where they are through localised approaches [44, 47, 50, 53, 59].

#### Culture-centred approaches

For people’s lived experiences of marginalisation to be included in NCD prevention policies, studies frequently reported a *culture of collaboration and allyship (sub-theme 5)* as essential. Such cultures were underpinned by mutual respect and benefits [40, 42, 44–46, 48], openness [42, 45, 46, 48, 58], humility [40, 50] and power sharing by questioning expert positioning [36, 37, 40–42, 46, 47, 50, 57]. A focus on building nonhierarchical relationships though effective communication [37, 39–42, 44, 46–48, 50, 52, 54, 56–59], flexible approaches [37, 49, 57] and teamwork [44, 49] enabled researchers to collaborate and serve as allies with communities. Community collaborations and allyship often required pre-existing relationships with communities [39, 41, 44, 49, 51, 53, 57] – a smaller number of quality relationships were reportedly more effective than a large quantity of relationships – and an understanding of and respect for differences in practice [37, 40, 43, 45–48, 57]. Studies indicated that collaboration and allyship-centred approaches to engaging people who are most likely to be affected by social inequities provided informal or formal support networks [36, 39, 40, 44, 46, 50, 56], reduced isolation [36, 39, 44, 56], and increased awareness and discussion of NCD prevention [36, 37, 40, 41, 44, 49–51, 53, 55–58, 60].

There was a major focus on incorporating lived experience in research to reduce inequities experienced by Black, Hispanic and multicultural communities (54% of studies) and First Nations peoples (22% of studies); five studies worked with First Nations peoples in the USA, four in Canada, and two in Australia. *Culturally safe and appropriate actions and spaces (sub-theme 6)* were core to including the experiences of these communities across the reviewed articles [39, 40, 43, 46, 50, 53]. Cultural safety was described in studies as respecting and understanding different cultural practices and knowledge systems [37, 40, 43, 45, 47, 48] and critically reflecting on the ways dominant cultures, values and beliefs influence power and interactions [61]. This includes recognising that the historical and ongoing oppression of non-Western practices and knowledge systems has contributed to community distrust of research [36, 37], which has been exacerbated by the failure of previous community-based research to meaningfully improve health and wellbeing for participants and communities. Owing to the numerous tensions between research and community interests, studies found that a commitment to *ongoing reflexivity and positionality (sub-theme 7)* was essential when working with communities to reduce inequities in NCDs [37, 39, 40, 45–47, 49, 50, 57, 59].

#### Tensions with existing institutional practices

The reviewed studies found that involving lived experience in research and policy was constrained by *tensions regarding dominant policy approaches (sub-theme 8)* [36, 37, 39, 40, 42–47, 49–51, 53, 55, 57]. Tensions included the ongoing impacts of conservative, neoliberal and Western policies that have historically oppressed certain population sub-groups and resulted in policy processes that are disconnected with the lived experiences of people experiencing marginalisation [37, 40, 45, 46, 50]. Several studies described how incorporating lived experiences of marginalisation can challenge power imbalances where diverse voices are typically erased or silenced [37, 40, 43, 46, 47, 50]. Indeed, policy impacts were reportedly dependent on the authorising policy environment and government leaders’ appetite for challenging power imbalances [36–38, 40, 58].

*Partnership and research tensions (sub-theme 9)* were frequently identified across studies and generally pertained to competing priorities and interests amongst partners [36–40, 44, 57, 62]. Researchers noted the need to minimise the burden of lived experience work on community partners when it was research-led, especially considering the research fatigue and trauma that has been experienced by many population groups (particularly First Nations peoples) [37, 39, 57]. Nevertheless, researcher-led approaches were at odds with the reoccurring notions of shared responsibility [37, 40, 49, 54] for including lived experience in NCD prevention policy and that researchers should mainly provide technical support roles to communities (e.g. analytical) and allow communities to direct the research process [37, 40, 49, 51, 54, 57]. Moreover, studies reported that lived experience research required spending significant time with communities [37, 38, 40, 44, 46–48, 50, 52, 55–58, 63]; a genuine commitment to co-learning and co-creating ideas across the whole project or policy cycle [40, 42, 44–46, 50, 52]; training community members [36–42, 44, 46–49, 52, 53, 56, 60]; and focusing on having direct impact and change [41, 49]. Adequate reimbursement to community members for their contributions as experts was also noted as important and frequently provided, though an absence of consistent and best practice reimbursement rates was evident across literature [37, 40–42, 59]. *Resource tensions (sub-theme 10)* were discussed in relation to the money and time required to meaningfully engage people who are most affected by social inequity in NCD prevention policy research, as resources were limited by most existing institutional structures (e.g. short-term research grants) [36–38, 42, 44, 48, 49, 52, 53, 56, 58].

#### Co-creating mechanisms for impact

The reviewed studies suggested that it was essential to *create networks of influence (sub-theme 11)* for the voices of people with lived experience of social marginalisation to impact NCD prevention policymaking. Networks of influence were defined as those that connect diverse stakeholders – with studies suggesting that relationships be established with government representatives early on, especially local government representatives who were viewed as accessible partners [40, 41, 44, 47, 50, 53, 56]. Studies indicated that these networks and relationships could then be strengthened through coalitions [36, 41, 42, 44, 48–50, 52, 54, 58]; community advisory committees [39, 40, 42, 45, 52, 54]; community engagement and outreach activities [36, 40, 41, 44, 47, 49, 57]; engaging community members or organisations as co-researchers [40–42, 44, 46, 49, 50, 53–55, 59]; and investing in community training and development [36–42, 44, 46–49, 52, 53, 56, 60].

Co-creation of NCD prevention policy ideas was identified as a core component of engaging people’s lived experiences in research, policy and practice [40, 42, 47, 49, 53, 55, 60]. Such co-creation was perceived to necessitate *safe spaces and ongoing opportunities for listening (sub-theme 12)* [39, 40, 43, 46, 50, 53, 59], *support self-determination (sub-theme 13)* [36–38, 40, 42, 46, 50, 54–56], *and focus on asset or strengths-based solutions (sub-theme 14)* [37, 40, 42, 45, 50]. In addition to traditional research methods (i.e. interviews, focus groups, and photovoice), storytelling [37, 40, 45, 48] and community deliberative forums [37, 39–41, 45, 56] were used to create safe listening spaces and support self-determination. A few other *innovations (sub-theme 15)* for maximising policy impact were explored across several studies and included digital advancements (e.g. online data platforms to reduce isolation for rural communities) [19, 42, 44, 47, 48, 52, 53, 59, 64] and community governance agreements (e.g. agreements on models of community partnerships) [36–38].

## Discussion

### Summary of findings

Our systematic scoping review of 49 studies is a novel attempt to understand research approaches for informing equitable NCD prevention policy through the lived experiences of people who are harmed by systems of marginalisation. We found that research has mostly engaged people’s lived experiences on the basis of their multicultural ethnicity (52% of studies), rural location (37%) and First Nations identification (22%). Two thirds of this evidence-base stems from the USA and is focused on preventing dietary risks (35% of studies) or multiple NCD risk factors (20%). Yet, in most high-income countries, gaps remain in our understanding of how the experiences and voices of population groups who experience discrimination due to their sexuality, gender and/or disability can be included in NCD prevention policy – particularly as it pertains to mental health and alcohol use. There is also scope to better understand how diverse groups of young people can be included in research to inform NCD prevention policy.

Although some of the reviewed evidence identified that the voices of people with lived experience can be engaged to inform state and federal level policy, studies were typically focussed on policy advocacy to local governments, often supported by local coalitions. Our thematic synthesis indicated that policy-relevant research can be strengthened through co-creation of impact using networks of influence, safe listening spaces, self-determined and strengths-based approaches. In addition, the purpose of engaging community lived experience should be defined from the beginning to avoid tokenism; a culture-centred approach should be employed to strengthen collaboration, allyship, reflexivity and cultural safety; and tensions with existing institutional practices are likely to surface and need to be addressed (Fig. 4). Below we discuss these findings in relation to the broader literature and their implications for research, policy and practice.

### Research approaches to include lived experience in NCD prevention policy

Where community lived experiences have been sought in NCD prevention research, consultation processes are more common than community-led approaches where power is shared in decision-making. According to Arnstein’s Ladder of citizen participation, these are forms of nonparticipation or tokenism, which lack the partnerships, delegated power and citizen control for community members to have a genuine influence on policy decisions [25]. For example, in a systematic review of youth obesity prevention in diverse communities, only 9% of 126 studies included community-led approaches [65]. We add to this by identifying how NCD prevention research that engages lived experience typically only results in policy advocacy at the local government level (68% of reviewed studies). This finding potentially highlights that people who experience marginalisation are mostly being engaged to share their voices and experiences in spaces where power sharing is limited. Arnstein’s Ladder of citizen participation is a notable framework that public health researchers and policy stakeholders could become more aware of. Nonetheless, it is not without its limitations. These include its lack of validation with culturally diverse communities beyond America and limited empirical foundations, a hierarchical and linear policy understanding that overlooks other collective impact approaches, inadequate consideration of reflexivity and positionality, and limited community involvement in the research [66–68]. These limitations should be acknowledged and addressed in any future public health applications of Arnstein's model.

There have also been longstanding calls from the World Health Organization for Member States to adopt national evidence-based NCD prevention policies [69] and United Nations (UN) conventions and declarations outlining the rights of people experiencing marginalisation to contribute to policy decisions [17, 70]. Despite these national commitments, our findings suggest it is less clear if and how grassroots advocacy stemming from research can trigger systemic changes in high-level NCD prevention policy. Nonetheless, we found studies in the USA and Canada (*n* = 4 studies) that demonstrated how the voices of rural First Nations peoples [60, 71], First Nations and African American peoples [72], and women on low incomes [73] were included in research to inform national policy, mostly to address food insecurity. These studies were unique in their approaches, which included government directly intending to share power with Inuit organisations through policy actions [60]; continuous engagement with food justice coalitions, activists and collaboratives [71, 72]; and having a direct focus on achieving multiple policy advocacy outcomes [73]. Yet, Australian research has found that real opportunities for First Nations people to participate in healthy policy are limited, and informal interconnected networks are ultimately key for driving equitable changes [74]. Examples from Mexico and Chile have also shown the importance of civil society, coalitions and embedding community voice in advocacy for national obesity prevention policies [75]. Indeed, 22 of the included studies (45%) used coalitions to drive participation in the policy process. A previous systematic review of 26 studies of initiatives addressing the social determinants of health further showed the effectiveness of coalitions to be related to community context, resourcing, structuring, membership, partnerships, and planning, among other factors [76].

The use of theory was generally inconsistent across literature, with considerable scope to advance the inclusion of policy and CBPR theory and develop more culturally relevant and intersectional approaches to research focused on NCD prevention policy. In particular, our finding that the lived experiences of women, gender, sexual, and disability minority groups were underexplored in relation to NCD prevention policy (*n* = 5 studies) indicates the need to advance public health research by incorporating an intersectionality lens [77]. For example, applying an intersectionality lens to a social determinant understanding of health inequity would help recognise that disability is an experience that can intersect with other identities that experience discrimination (e.g. First Nations peoples, rural women and people on low incomes) [78, 79]. Future research should be seeking to better represent and understand these intersectional experiences of discrimination in NCD prevention policy with a view to comprehensively challenge structures of power and oppression, promote inclusive policy advocacy and change, and build cohesion and effectiveness across efforts to reduce health inequities. The USA stood out as the country leading in the application of such research methodologies, possibly because it is where the concepts of intersectionality and critical race theory have been espoused by scholars such as Kimberlé Crenshaw in the 1970 s, stemming from Black civil rights and feminist movements [77].

In addition, it will be important to strengthen the inclusion of people from low- and middle-income countries in NCD prevention and global health policy and research. To ensure studies in our review were comparable across countries with similar NCD, equity, research and policy priorities, we did not include research from low- and middle-income countries. It is becoming increasingly recognised that there should be less emphasis on high-income country supremacy in global health and more investment in developing local leaders and local solutions in low- and middle-income countries [80]. To facilitate this, researchers from low- and middle-income countries have called for improved research infrastructure, partnerships with researchers and practitioners who have lived experience, intellectual property agreements, and more equitable distribution of research funds [80].

### Factors affecting the inclusion of lived experience in NCD prevention policy

Our review indicated that a range of methods can be used to understand and include people’s lived experiences in NCD prevention policy research. Whilst no one method can be considered best practice, our thematic analysis and the existing literature suggest that there are certain factors that enable the success of projects aiming to incorporate lived experience in NCD prevention policy [81]. Our findings extend previous research focused on strengthening Aboriginal and Torres Strait Islander experiences and voices in health policy [82]. Using policy theory and interviews with policy actors, researchers in Australia proposed a framework for First Nations health advocacy through a combination of Aboriginal and Torres Strait Islander leadership, research evidence, strategic storytelling, and coordinated advocacy coalitions [82]. Qualitative evidence from Canada also sought to understand how First Nations peoples could be meaningfully involved in health policy via interviews with First Nations and policy leaders [83]. The resulting RIPPLES framework included elements of recognition and representation, interrupting and re-imagining relationships, preparing agreements for working relationships, practising protocols on how people will work together, leveraging power to influence decisions, exerting community authority, and shifting social structures and barriers to First Nations involvement. Another example from Canada is the Our Health Counts: In Our Voices project, which co-designed and respectfully implemented self-determined health surveys in urban First Nations populations to address data gaps and ultimately increase resources and program funds [84].

Our review supports the idea that the purpose of lived experience research in NCD prevention is not only to support community participation and culturally safe leadership but to also challenge power imbalances in decision-making, address the root causes of social inequities such as systems of marginalisation and inadequate distribution of resources, and facilitate community healing [85, 86]. Indeed, efforts to better represent the voices of people experiencing marginalisation in NCD prevention policy continue to be constrained by inadequate policy actions that dismantle the systemic root causes of these inequities (i.e. racism, colonisation, neoliberalism, sexism, ablism, among others) [1]. The need for ongoing structural policy reform to equitably improve population health is coupled with the need to be conscious of the trauma and othering that communities have experienced from existing policies, structures and societal events [87, 88]. Although research on trauma-informed approaches in public health have not been widely researched in Western circles, much can be learnt from First Nations approaches to healing, such as *Dadirri* – inner, deep listening to one’s self and spirit – developed by Aboriginal communities in Australia [87]. *Dadirri* has been shown to “reconfigure futures, participants and researchers” by breaking down oppressive research practices to allow people to own the narratives of their lives [87].

Whilst culturally appropriate NCD prevention initiatives is not a new concept [81], we found evidence that this needs to be better combined with cultures of allyship and support for self-determination. Allyship moves beyond traditional notions of community partnership and engagement [81] towards reflecting on our own positions of power and privilege through positionality and reflexivity, and making space to proactively advocate for the rights and self-determination of underrepresented communities [89–91]. If we cannot create these spaces, there is a risk that oppressive systems will continue to drive NCD prevention research and policy, and communities will experience re-traumatisation.

Further, our review shows that efforts to challenge power imbalances in decision-making by elevating people’s voices and experiences of marginalisation are likely to be met by resistance to change from powerful institutions that have vested interests in maintaining the status quo. These findings are similar to the barriers reported in a previous review that examined community engagement approaches to improving the health and wellbeing of populations, particularly people who experience marginalisation, in the UK [92]. As we found, community partnerships are limited if research and other institutions are not committed to power sharing, have poor relationships with communities historically harmed by dominant systems, have competing priorities and/or goals, and resources and investments are not made to spend time with – and ultimately support capacity building among—communities [92]. To address these tensions and drive equitable NCD prevention policy, our analysis of 49 studies indicates that co-creating culturally appropriate mechanisms for impact with communities is likely to be important, although the efficacy of such co-creation needs to continue to be evaluated.

### Strengths and limitations

To the best of our knowledge, this is a novel synthesis of evidence exploring how people experiencing marginalisation can be included in research aiming to inform NCD prevention policy development in high-income settings. Our scoping review methodology enabled us to review more than 10 000 records retrieved from six databases. However, our findings are limited to only a few populations from high-income countries, and additional work is needed to synthesise this knowledge in countries beyond the USA and in low- and middle-income settings. Another limitation of our study was that we could not identify efforts that have not been evaluated, published or reported to a high enough academic standard, nor those published in languages other than English. As such, better reporting standards and monitoring of such evidence is required. In addition, whilst representation across the authorship groups was not examined, it is known that studies often do not include the voices of the people being studied in their design [45, 64, 72], which is a priority in First Nations and culturally safe research processes [93, 94].

### Conclusions and implications for future research, policy and practice

Through this review we explored research approaches that have been used to engage people’s lived experience to inform equitable NCD prevention policy. We identified 15 areas, within four domains, that should be addressed to potentially strengthen these research processes. Researchers and policy leaders will need to continue to collaborate with diverse communities to identify and co-design best practice mechanisms that support community power in NCD prevention policy development, including localised approaches in low-, middle- and high-income countries. That is, research could be carried out in ways that prioritise allyship with communities, for example, by creating opportunities for communities to lead and influence research and policy decisions and more equitably access resources and funds. Ongoing engagement with positionality and reflexivity will also be necessary to deconstruct traditional public health research and policy principles, philosophies and practices that seldom acknowledge and amplify diverse lived experiences and community power.

## Supplementary Information

Additional file 1.

## Data Availability

See supplementary material.

## Abbreviations

CASP: Critical Appraisal Skills Programme
CINAHL: Cumulative Index to Nursing and Allied Health Literature
NCD: Non-communicable disease
OECD: Organisation for Economic Co-operation and Development
PRISMA: Preferred Reporting Items for Systematic Reviews and Meta-Analyses
UK: United Kingdom
UN: United Nations
USA: United States of America
