# Supplementary material for: Informing equitable noncommunicable disease prevention policies through lived experience: a scoping review of research approaches
Source: Health Res Policy Syst. 2025 Nov 27;23:155. doi: 10.1186/s12961-025-01348-2 (PMC12661748; doi:10.1186/s12961-025-01348-2)
Supplement: Supplementary file 1 — Additional file 1. [file 12961_2025_1348_MOESM1_ESM.docx]

**Supplementary Information**

***Informing equitable NCD prevention policies through lived experience: A scoping review of research approaches***

**Table S1.** Medline Search Strategy (February 2022)

| Social disadvantage | - disadvantage* - marginali* - vulnerab* N20 health* - equit* - inequalit* - depriv* - disparit* - education* N5 low* - income* N5 low* - wage* N5 low* - occupation* N5 low* - employ* N5 low* - job* N5 low* - “social class*” - “social hierarch*” - “social status” - “social position” - “socioeconomic position” - “socio-economic position” - SEP - “socioeconomic status” - “socio-economic status” - SES - socio-economic N20 health* - socioeconomic N20 health* - ethnic* N20 health* - race* N20 health* - raci* - minorit* - CALD - “cultural* and linguistic* divers*” - “Limited English Proficienc*” - language* N20 health* - refugee* - migrant* - “asylum seeker*” - asylum-seeker* - disabilit* - disabled - rural* N20 health* - residence - poverty - (Indigen* OR Aborigin* OR “Torres Strait Island*” OR “First People*” OR Maori* OR “First Nation*” OR Inuit* OR Metis OR “American Indian*” OR Amerindian* OR Eskimo* OR “Native American*” OR “Native Canadian*” OR “Native Hawaiian*” OR “Native people*” OR “Native population*” OR Tribal OR Tribe* OR (Native* N2 Alaska*) OR “North American Native” OR (Native* N2 Siberia*) OR Saami OR Sami OR Greenlandic* OR Nunavut* OR (Indian* NOT (India or India’s or India/) OR ((Remote OR Isolated OR Northern) N3 Communit*) OR (“New Zealand” AND ethnic*)) - Gender* N5 (inequalit* OR unequal OR divers* OR identit* OR expression* OR non-conform* OR nonconform OR gap OR based OR income* OR wage* OR education* OR occupation* OR work* OR employ* OR job*) - sex* N5 (inequalit* OR unequal OR divers* OR orientation OR expression OR income* OR wage* OR education* OR occupation* OR employ* OR job*) - women* N5 (inequalit* OR unequal OR divers* OR income* OR wage* OR education* OR occupation* OR work* OR employ* OR job*) - LGBT* OR gay* OR lesbian* OR bisexual* OR “trans” OR transgender* OR transphobi* OR transsex* OR intersex* OR queer* OR homophobi* OR biphobi* OR SOGIE OR non-binary OR nonbinary OR homosex* OR “same sex” - (MH "Health Status Disparities") - (MH "Health Disparity, Minority and Vulnerable Populations+") - (MH "Socioeconomic Factors+") - (MH "Gender Equity") - (MH "Health Equity") - (MH "Social Marginalization") - (MH "Social Deprivation") - (MH "Cultural Deprivation") - (MH "Native Hawaiian or Other Pacific Islander") OR (MH "Indigenous Canadians+") - (MH "Indigenous Peoples") |
| --- | --- |
| AND  Lived experience | - “lived experience*” - “life experience*” - story* - stories - narrative* - voice* - perspective* - opinion* - phenomenolog* - ethnograph* - qualitative* - “critical theory” - (MH "Social Theory+") - (MH "Qualitative Research+") - (MH "Anthropology, Cultural+") - (MH "Narration+") |
| AND “Citizen participation” | - (citizen* OR public OR communit* OR stakeholder* OR civil*) N5 (participat* OR power* OR control* OR inform* OR educat* OR manipulat* OR engag* OR involve* OR consult* OR collaborat* OR empower* OR integrat* OR mobilis* OR mobiliz* OR collaborat* OR jury OR juries OR forum* OR consortium* OR coalition* OR union* OR alliance* OR associat* OR federation* OR network*) - advisory N2 (group* OR board* OR committee* OR panel* OR role* OR council*) - “citizen centred” - “delegated power” - “people power” - partnership* - placation - tokenis* - “participatory research” - “participatory action” - co-creat* - cocreat* - co-produc* - coproduc* - co-design* - codesign* - co-learn* - colearn* - outreach - activis* - champion* - “joint ventur*” - (MH "Stakeholder Participation+") - (MH "Social Participation") - (MH "Community Participation+") |
| AND  NCDs and their risk factors (including the social determinants of NCDs) | - “noncommunicable disease*” - “non-communicable disease*” - NCD* - cancer* - malignan* - tumour* - carcinoma* - “cardiovascular disease*” - CVD - “heart disease*” - Cardiac - Coronary - “chronic respiratory disease*” - “chronic obstructive pulmonary disease*” - COPD - “lung disease*” - “pulmonary hypertension” - Diabetes - “high blood sugar” - “high blood glucose” - hyperglycemia - hyperglycaemia - mental N4 (health OR illness* OR distress OR disorder* OR condition*) - “preventive health” - “preventative health” - “primary prevention” - Food* - Nutrition* - diet* - beverage* - Alcohol* - Liquor* - Obes* - Weight - “body mass index” - BMI - Tobacco - Cigarette* - Nicotine - “drug use” - drug* N5 use* - addict* - smoking - smoke* - “physical activity” - “physical inactivity” - exercise - environment* N5 health* - “built environment*” - “physical environment*” - “urban health*” “social determinant*” - “commercial determinant*” - “political determinant*” - “living condition*” - “working condition*” - “structural determinant*” - “material circumstance*” - labour N2 (market* OR system OR policy OR policies) - education N2 (system* OR policy OR policies) - health N2 (system* OR policy OR policies) - public N2 (system* OR policy OR policies) - (“social protection” OR welfare) N2 (system* OR policy OR policies) - “urban plan*” OR “urban policies” OR “urban policy” - “economic policy” OR “economic policies” - “social policy” OR “social policies” |
| AND  Policy outcomes | - policy N2 (impact* OR inform* OR develop* OR creat* OR advis* OR influence* OR shape* OR make* OR making) - policymak* - “policy advocacy” - “policy advocate*” - “politic* will*” - “policy commit*” - “politic* commit*” - “decision making” - “strategy development” - policy N5 (strateg* OR plan* OR intervention*) - government* N5 (policy OR policies OR strateg* OR plan* OR intervention* OR service* OR system*) - national N5 (policy OR policies OR strateg* OR plan*) - state N5 (policy OR policies OR strateg* OR plan*) - municipal* N5 (policy OR policies OR strateg* OR plan*) - local* N5 (policy OR policies OR strateg* OR plan*) - council* N5 (policy OR policies OR strateg* OR plan*) - department* N5 (policy OR policies OR strateg* OR plan*) - “public service*” - governance - coalition* |

**Table S2.** An overview of studies (n=49) that have incorporated priority populations’ lived experiences in NCD prevention policies by governments in high-income countries.

|  | **Policy focus** | **Country (locality); Indicators of marginalisation** | **Youth (yes/no)** | **Study design; Research methods** | **Policy cycle stage** | **Level of policy impact** |
| --- | --- | --- | --- | --- | --- | --- |
| Alkon et al., (2009) (1) | Healthy food access | USA (Karuk Tribe of California and West Oakland Food Collaborative); First Nations; Black community | no | Case study; Interviews (n=n/a); Observation | Policy evaluation; Policy advocacy | Local; Federal |
| Chilton et al., (2009) (2) | Food insecurity | USA (Philadelphia); Gender; Low income | no | Qualitative; Photovoice; Interviews (n=42 recruited from previous research at a local hospital) | Policy advocacy | Federal; State |
| Jernigan et al., (2011) (3) | Environmental determinants of healthy eating and active living | USA (Round Valley, California); First Nations; Rural | no | Qualitative case study; Focus Groups; Interviews (n=40 recruited through local leaders) | Policy advocacy | Local; Community |
| Lardeau et al., (2011) (4) | Food insecurity | Canada; First Nations; Rural | no | Qualitative; Photovoice (n=8 recruited from food bank, soup kitchen, and cultural centre) | Policy advocacy | Local |
| McClymont Peace et al., (2012) (5) | Food insecurity | Canada (Yukon, Northwest Territories, Ontario); First Nations; Rural | no | Qualitative case study; Focus group; Visual methods (n=150 recruited via First Nations organisations) | Policy advocacy | Federal |
| Wakegijig et al., (2013) (6) | Food insecurity | Canada (Nunavut); First Nations; Rural | no | Qualitative case study; Focus groups; consensus panel (n=n/a) | Policy formulation | Federal; State |
| Dutta et al., (2013) (7) | Food insecurity | USA (Indiana); Low income | no | Qualitative; Photovoice; Interviews; Focus groups (n=18 food pantry users) | Policy advocacy | Local; Community |
| Fillion et al., (2014) (8) | Food insecurity | Canada (Inuvialuit Settlement Region, Northwest Territories); First Nations; Rural | no | Qualitative; Focus groups (n=23 recruited from community organisations, local and territorial governments and universities) | Policy advocacy | Local |
| Sadler et al., (2014) (9) | Food policy | USA (Flint, Michigan); Low income; Black community | no | Qualitative; Observation; Interviews (n=30 recruited through food policy council) | Policy advocacy | State; Local |
| Akom et al., (2016) (10) | Environmental determinants of healthy eating and active living | USA (East Oakland); Low income; Black, Hispanic and multicultural communities | yes | Qualitative case study; Visual methods (n=90 recruited form youth and school partnerships) | Policy advocacy | Local |
| McCartan et al., (2016) (11) | Food environment | Australia (West Victoria); Rural | no | Qualitative case study; Interviews (n=11 members of food policy coalition) | Policy advocacy | Local |
| Johnson et al., (2017) (12) | Healthy eating | USA (Baltimore city); Ethnicity; Low income | yes | Qualitative; Photovoice (n=17 youth from local partners and a summer youth camp) | Policy advocacy | Local |
| Fehring et al., (2019) (13) | Reduce SSB consumption | Australia (Cape York, Queensland); First Nations; Rural | no | Case study; Interviews (n=37 recruited from existing community partners) | Policy evaluation | Local |
| Monteith et al., (2019) (14) | Food insecurity | Canada (Nova Scotia); Gender | no | Qualitative evaluation; Interviews (n=7 involved in participatory food costing) | Policy advocacy; Policy implementation | Local; All Government levels |
| Weinstein et al., (2019) (15) | Healthy eating and weight | USA (Philadelphia); Disability; Homelessness; majority Black participants | no | Qualitative; Focus group; Photovoice (n=8 recruited through housing partner and previous study involvement) | Policy advocacy | Local |
| Gravina et al., (2020) (16) | Food environment | Spain (Bilbao); Low income |  | Qualitative; Photovoice (n=9 recruited using traditional methods and supported by community partners) | Policy advocacy | Local |
| Freedman et al., (2022) (17) | Equitable food systems | USA (Cleveland, Ohio); majority Black; Low income | no | Mixed methods; Systems mapping (n=330 recruited through known partners) | Policy evaluation | Local; Community |
| Duffy et al., (2010) (18) | Social Determinants of Health | Canada (Moncton); Gender; Single mothers | no | Qualitative; Photovoice (n=7 recruited via community partner) | Policy advocacy | Local; community |
| Kramer et al., (2010) (19) | Healthy eating and active living | USA (Colorado); Majority Hispanic and Black community; Low income | yes | Qualitative; Photovoice (n=50, broad community involvement) | Policy advocacy; Policy adoption | Local |
| Young et al., (2010) (20) | CVD prevention | Canada (Ontario, British Columbia) and USA (Washington); Low income; Gender | yes | Mixed methods; Focus groups; Interviews (n=38 recruited via community partners) | Policy advocacy | All government levels |
| Collie-Akers et al., (2013) (21) | CVD and diabetes prevention | USA (Kansas); Low income; Hispanic community | no | Pre/post; Consensus panel (n=10 recruited by community and coalition partners) | Policy implementation; Policy evaluation | Local |
| Fawcett et al., (2013) (22) | Healthy lifestyles for chronic disease prevention | USA (Kansas); Low income; Hispanic community | no | Qualitative case study; Interviews (n=n/a coalition members and partners) | Policy implementation | Local; Community |
| Madrigal et al., (2014) (23) | Healthy environments | USA (Salinas); Hispanic community; Agricultural areas | yes | Qualitative; Photovoice (n=15 recruited via community partners) | Policy advocacy | Local |
| Street et al., (2018) (24) | Healthy lifestyles for children | Australia (South Australia); First Nations; Rural | no | Qualitative; Focus group; Co-create (n=14 recruited via community social media group and word of mouth) | Policy advocacy | All government levels; Community |
| Kelly et al., (2019) (25) | Diabetes prevention | USA (Inkster, Michigan); Ethnicity unclear; Low income | no | Qualitative case study; Focus group; Interviews (n=XX from partnerships) | Policy formulation; Policy evaluation | Local; Community |
| Agner et al., (2020) (26) | Healthy environments | USA (Hawaii); Low income; Rural; Native Hawaiian and multicultural communities | no | Qualitative; Interviews; Visual methods (n=10 key informants who were aware of initiative) | Policy evaluation; Policy agenda setting | State |
| Walker et al., (2022) (27) | Equitable chronic disease prevention | USA (Pierce County, Washington); Rural; Black, Hispanic, biracial, American Indian, Native Hawaiian and Pacific Islander communities | yes | Qualitative case study; Focus groups (n=214 recruited via email, text and virtual flyers) | Policy formulation | Local |
| Findholt et al., (2010) (28) | Obesity prevention | USA (Union County, Oregon); Rural | yes | Qualitative; Photovoice (n=6 recruited from high school) | Policy advocacy | Local |
| Kramer et al., (2013) (29) | Obesity prevention | USA (California - Santa Rose, West Modesto, Richmond; Colorado -Denver, Commerce City, Denver Urban Gardens); Hispanic and Black communities | yes | Qualitative; Photovoice (n=50) | Policy evaluation | Local; Community |
| Hackett et al., (2015) (30) | Obesity prevention | USA (New York State); Low income; Black and Hispanic communities | yes | Qualitative; Photovoice (n=9 recruited from youth-run farmers market) | Policy advocacy | Local |
| John et al., (2017) (31) | Obesity prevention | USA (6 Western states: Oregon, Washington, Nevada, Colorado, New Mexico, Idaho); Rural | yes | Qualitative; Visual methods; Focus group (n=600 recruited from schools, community organisations, and local government via community partner) | Policy advocacy | Local |
| Diez et al., (2018) (32) | Obesity prevention | Spain (Villaverde, Madrid); Low income | no | Qualitative; Photovoice (n=36 recruited using traditional methods and through neighbourhood associations) | Policy advocacy | Local |
| Campbell et al., (2004) (33) | Equitable mental health services | UK (England); African-Caribbean community | no | Qualitative case study; Interviews; Focus groups (n=30 local community stakeholders from across sectors) | Policy evaluation; Policy advocacy | State |
| Perez et al., (2016) (34) | Broad mental healthy policy | USA (North Carolina); Hispanic community | no | Qualitative; Photovoice (n=21 promotoras/lay health workers who has previously participated in the first study phase) | Policy advocacy | Local |
| Thirlwall et al., (2018) (35) | Public mental health strategy | UK (Dumfries and Galloway); Rural | no | Qualitative case study; Focus group (n=443 recruited from community services) | Policy formulation | Local; Community |
| Forenza et al., (2019) (36) | Community empowerment to address community stress | USA (northeaster urban locale); Black and Hispanic communities; Low income | yes | Qualitative; Focus group; Interviews (n=102 recruited from substance abuse prevention coalition and youth who are coalition service users) | Policy evaluation; Policy advocacy | Local; Community |
| Gullon et al., (2010) (37) | Physical activity environment policy | Spain (Madrid) | no | Qualitative; Photovoice (n=55 recruited through flyers, meetings, community partners) | Policy advocacy | Local |
| Arredondo et al., (2013) (38) | Physical activity policy | USA (San Diego); Hispanic community; Low SEP area | yes | Mixed methods; observation; visual methods (n=9 recruited by community leaders) | Policy advocacy; Policy implementation | Local |
| Mier et al., (2013) (39) | Physical activity policy | USA; Hispanic community; Low income | yes | Qualitative; Focus groups (n=67 recruited from stakeholder meetings) | Policy advocacy; Policy agenda setting | Local |
| Choy et al., (2016) (40) | Physical activity policy | USA (Hawaii); Rural | no | Qualitative case study; Interviews (n=25 stakeholders involved in the initiative) | Policy evaluation; Policy adoption | State; Local |
| Winter et al., (2016) (41) | Physical activity policy | USA (California) | yes | Mixed methods; Photovoice; Ethnicity (n=20 citizen scientists) | Policy advocacy | Local |
| Rydenstam et al., (2020) (42) | Physical activity policy | Sweden (V¨asterås); Low income | yes | Qualitative; Photovoice (n=24 recruited from local schools and community centres) | Policy advocacy | Local |
| Struthers et al., (2003) (43) | Smoke-free policy | USA (Indian reserves in South Dakota, Minnesota, and Nebraska); First Nations; Rural | no | Qualitative; Geospatial methods; Observation (n=n/a) | Policy agenda setting; Policy evaluation | Local; Community |
| Mahon et al., (2007) (44) | Smoke-free policy | USA; Rural | no | Qualitative case study; Interviews; Observation (n=n/a) | Policy advocacy | Local |
| Tanjasiri et al., (2013) (45) | Tobacco control policy | USA (California and Washington); Asian American and Pacific Islander | yes | Mixed methods; Geospatial methods; Photovoice | Policy advocacy | Local |
| Petteway et al., (2019) (46) | Smoke-free policy | USA (Southwest Baltimore); Low income | yes | Qualitative; Photovoice (n=14 from schools) | Policy advocacy; Policy adoption | Local |
| Kingsbury et al., (2020) (47) | Tobacco control policy | USA (Minnesota); Black communities | yes | Qualitative; Interviews (n=16 via partner organisations) | Policy advocacy; Policy adoption | State; Local |
| Herd et al., (2011) (48) | Alcohol | USA (7 major cities); Black communities | no | Qualitative; Interviews (n=184 recruited through community leaders) | Policy advocacy; policy evaluation; | State; Local |
| Drabble et al., (2014) (49) | Alcohol | USA (Oakland, Los Angeles, Milwaukee, Wisconsin, San Antonio, Raleigh, Detroit, Baltimore); Black communities | no | Qualitative; Interviews (n=184 advocates recruited via snowball sampling) | Policy formulation; policy implementation | State; Local |

**Table S3.** Critical appraisal results

|  | **Q1. Research design** | **Q2. Research design** | **Q3. Data collection** | **Q4. Data collection** | **Q5. Data collection** | **Q6. Data analysis** | **Q7. Data analysis** | **Q8. Data analysis** | **Q9. Research findings** | **Q10. Research findings** | **Total score** |
| --- | --- | --- | --- | --- | --- | --- | --- | --- | --- | --- | --- |
| **Dietary Risks** | | | | | | | | | | | |
| Alkon et al., (2009) | **🗶** | **✓** | **✓** | **?** | **✓** | **✓** | **?** | **?** | **?** | **?** | 4/10 |
| Chilton et al., (2009) | **✓** | **✓** | **✓** | **✓** | **✓** | **🗶** | **✓** | **🗶** | **✓** | **✓** | 8/10 |
| Jernigan et al., (2011) | **✓** | **✓** | **✓** | **✓** | **✓** | **✓** | **✓** | **✓** | **✓** | **✓** | 10/10 |
| Lardeau et al., (2011) | **✓** | **✓** | **✓** | **✓** | **✓** | **🗶** | **✓** | **✓** | **✓** | **✓** | 9/10 |
| McClymont Peace et al., (2012) | **✓** | **✓** | ? | **✓** | **✓** | **🗶** | **✓** | **🗶** | **✓** | **✓** | 7/10 |
| Wakegijig et al., (2013) | **✓** | **✓** | **✓** | **🗶** | ? | **🗶** | ? | **🗶** | **✓** | **✓** | 5/10 |
| Dutta et al., (2013) | **✓** | **✓** | **✓** | **✓** | **✓** | **🗶** | **✓** | **✓** | **✓** | **✓** | 9/10 |
| Fillion et al., (2014) | **✓** | **✓** | **✓** | **🗶** | **✓** | **🗶** | **✓** | **🗶** | **✓** | **✓** | 7/10 |
| Sadler et al., (2014) | **✓** | **✓** | **✓** | **✓** | **✓** | **🗶** | ? | **✓** | **✓** | **✓** | 8/10 |
| Akom et al., (2016) | **✓** | **✓** | **✓** | **✓** | **✓** | **🗶** | ? | **✓** | **✓** | **✓** | 8/10 |
| McCartan et al., (2016) | **✓** | **✓** | **✓** | **✓** | **✓** | **🗶** | **✓** | **✓** | **✓** | **✓** | 9/10 |
| Johnson et al., (2017) | **✓** | **✓** | **✓** | **✓** | **✓** | **🗶** | **✓** | **✓** | **✓** | **✓** | 9/10 |
| Fehring et al., (2019) | **✓** | **✓** | **✓** | **✓** | **✓** | **🗶** | **✓** | **✓** | **✓** | **✓** | 9/10 |
| Monteith et al., (2019) | **✓** | **✓** | **✓** | **✓** | **✓** | **🗶** | **✓** | **✓** | **✓** | **✓** | 9/10 |
| Weinstein et al., (2019) | **✓** | **✓** | **✓** | **✓** | **✓** | **🗶** | **✓** | **✓** | **✓** | **✓** | 9/10 |
| Gravina et al., (2020) | **✓** | **✓** | **✓** | **✓** | **✓** | **🗶** | **✓** | **✓** | **✓** | **✓** | 9/10 |
| Freedman et al., (2022) | **✓** | **✓** | **✓** | **✓** | **✓** | **🗶** | **✓** | **✓** | **✓** | **✓** | 9/10 |
| **NCD Prevention** | | | | | | | | | | | |
| Duffy et al., (2010) | **✓** | **✓** | **✓** | **✓** | **✓** | **🗶** | **✓** | **✓** | **✓** | **✓** | 9/10 |
| Kramer et al., (2010) | **✓** | **✓** | **✓** | ? | **✓** | **🗶** | ? | **✓** | **✓** | **✓** | 7/10 |
| Young et al., (2010) | **✓** | **✓** | **✓** | ? | ? | **🗶** | ? | ? | **✓** | **✓** | 5/10 |
| Collie-Akers et al., (2013) | **✓** | **✓** | **✓** | **🗶** | **✓** | **🗶** | **✓** | **✓** | **✓** | **✓** | 8/10 |
| Fawcett et al., (2013) | **✓** | **✓** | **✓** | ? | **✓** | **🗶** | ? | **✓** | **✓** | **✓** | 7/10 |
| Madrigal et al., (2014) | **✓** | **✓** | **✓** | **✓** | **✓** | **🗶** | **✓** | **✓** | **✓** | **✓** | 9/10 |
| Street et al., (2018) | **✓** | **✓** | **✓** | **✓** | **✓** | **✓** | **✓** | **✓** | **✓** | **✓** | 10/10 |
| Kelly et al., (2019) | **✓** | **✓** | **✓** | **🗶** | **✓** | **🗶** | ? | **✓** | **✓** | **✓** | 7/10 |
| Agner et al., (2020) | **✓** | **✓** | **✓** | **🗶** | **✓** | **🗶** | ? | **✓** | **✓** | **✓** | 7/10 |
| Walker et al., (2022) | **✓** | **✓** | **✓** | **✓** | **✓** | **🗶** | ? | **✓** | **✓** | **✓** | 8/10 |
| **Obesity prevention** | | | | | | | | | | | |
| Findholt et al., (2010) | **✓** | **✓** | **✓** | **✓** | **✓** | **🗶** | **✓** | **✓** | **✓** | **✓** | 9/10 |
| Kramer et al., (2013) | **✓** | **✓** | **✓** | ? | **✓** | **🗶** | ? | **✓** | **✓** | **✓** | 7/10 |
| Hackett et al., (2015) | **✓** | **✓** | **✓** | **✓** | **✓** | **🗶** | **✓** | **✓** | **✓** | **✓** | 9/10 |
| John et al., (2017) | **✓** | **✓** | **✓** | **✓** | **✓** | **🗶** | **✓** | **✓** | **✓** | ? | 8/10 |
| Diez et al., (2018) | **✓** | **✓** | **✓** | **✓** | **✓** | **🗶** | **✓** | **✓** | **✓** | **✓** | 9/10 |
| Campbell et al., (2004) | **✓** | **✓** | **✓** | ? | **✓** | **🗶** | ? | **✓** | **✓** | **✓** | 7/10 |
| Perez et al., (2016) | **✓** | **✓** | **✓** | **✓** | **✓** | **🗶** | **✓** | **✓** | **✓** | **✓** | 9/10 |
| Thirlwall et al., (2018) | **✓** | **✓** | **✓** | ? | ? | **🗶** | ? | **🗶** | **✓** | **✓** | 5/10 |
| Forenza et al., (2019) | **✓** | **✓** | **✓** | **✓** | **✓** | **🗶** | ? | **✓** | **✓** | **✓** | 8/10 |
| **Physical activity** | | | | | | | | | | | |
| Gullon et al., (2010) | **✓** | **✓** | **✓** | **✓** | **✓** | **🗶** | **✓** | **✓** | **✓** | **✓** | 9/10 |
| Arredondo et al., (2013) | **✓** | **✓** | **✓** | **✓** | **✓** | **🗶** | ? | ? | **✓** | **✓** | 7/10 |
| Mier et al., (2013) | **✓** | **✓** | **✓** | **✓** | **✓** | **🗶** | **✓** | **✓** | **✓** | **✓** | 9/10 |
| Choy et al., (2016) | **✓** | **✓** | **✓** | **✓** | **✓** | **🗶** | **✓** | **✓** | **✓** | **✓** | 9/10 |
| Winter et al., (2016) | **✓** | **✓** | **✓** | **✓** | **✓** | **🗶** | **✓** | **✓** | **✓** | **✓** | 9/10 |
| Rydenstam et al., (2020) | **✓** | **✓** | **✓** | **✓** | **✓** | **🗶** | **✓** | **✓** | **✓** | **✓** | 9/10 |
| **Tobacco** | | | | | | | | | | | |
| Struthers et al., (2003) | **✓** | **✓** | ? | **✓** | **✓** | **🗶** | ? | **✓** | **✓** | **✓** | 7/10 |
| Mahon et al., (2007) | **✓** | **✓** | **✓** | **🗶** | **✓** | **🗶** | **✓** | ? | **🗶** | ? | 5/10 |
| Tanjasiri et al., (2013) | **✓** | **✓** | **✓** | **🗶** | **✓** | **🗶** | **✓** | **✓** | **✓** | **✓** | 8/10 |
| Petteway et al., (2019) | **✓** | **✓** | **✓** | **✓** | **✓** | **🗶** | **✓** | **✓** | ? | **✓** | 8/10 |
| Kingsbury et al., (2020) | **✓** | **✓** | **✓** | ? | **✓** | **🗶** | **✓** | ? | **✓** | **✓** | 7/10 |
| **Alcohol** | | | | | | | | | | | |
| Herd et al., (2011) | ? | **✓** | **✓** | **✓** | **✓** | **🗶** | **🗶** | **✓** | **✓** | ? | 6/10 |
| Drabble et al., (2014) | **✓** | **✓** | **✓** | **✓** | ? | **🗶** | **🗶** | **✓** | **✓** | **✓** | 7/10 |

**✓=Yes; 🗶=No;** ?=**Can’t tell**

**References**

1. Alkon AH, Norgaard KM. Breaking the Food Chains: An Investigation of Food Justice Activism*. Sociological Inquiry. 2009;79(3):289-305.

2. Chilton M, Rabinowich J, Council C, Breaux J. Witnesses to hunger: participation through photovoice to ensure the right to food. Health Hum Rights. 2009;11(1):73-85.

3. Jernigan VB, Salvatore AL, Styne DM, Winkleby M. Addressing food insecurity in a Native American reservation using community-based participatory research. Health Educ Res. 2012;27(4):645-55.

4. Lardeau MP, Healey G, Ford J. The use of Photovoice to document and characterize the food security of users of community food programs in Iqaluit, Nunavut. Rural and remote health. 2011;11(2):1680.

5. McClymont Peace D, Myers E. Community-based participatory process--climate change and health adaptation program for Northern First Nations and Inuit in Canada. Int J Circumpolar Health. 2012;71(0):1-8.

6. Wakegijig J, Osborne G, Statham S, Issaluk MD. Collaborating toward improving food security in Nunavut. Int J Circumpolar Health. 2013;72.

7. Dutta MJ, Anaele A, Jones C. Voices of Hunger: Addressing Health Disparities Through the Culture-Centered Approach. 2013.

8. Fillion M, Laird B, Douglas V, Van Pelt L, Archie D, Chan HM. Development of a strategic plan for food security and safety in the Inuvialuit Settlement Region, Canada. Int J Circumpolar Health. 2014;73:25091.

9. Sadler R, Arku G, Gilliland J. Local food networks as catalysts for food policy change to improve health and build the economy. Local Environment: The International Journal of Justice and Sustainability. 2014;20(9):1103–21.

10. Akom A, Shah A, Nakai A, Cruz T. Youth Participatory Action Research (YPAR) 2.0: how technological innovation and digital organizing sparked a food revolution in East Oakland. Int J Qual Stud Educ. 2016;29(10):1287-307.

11. McCartan J, Palermo C. The role of a food policy coalition in influencing a local food environment: an Australian case study. Public health nutrition. 2017;20(5):917-26.

12. Johnson KA, Steeves EA, Gewanter ZR, Gittelsohn J. Food in My Neighborhood: Exploring the Food Environment through Photovoice with Urban, African American Youth. J Hunger Environ Nutr. 2017;12(3):394-405.

13. Fehring E, Ferguson M, Brown C, Murtha K, Laws C, Cuthbert K, et al. Supporting healthy drink choices in remote Aboriginal and Torres Strait Islander communities: a community-led supportive environment approach. Australian and New Zealand journal of public health. 2019;43(6):551-7.

14. Monteith H, Anderson B, Williams PL. Capacity building and personal empowerment: participatory food costing in Nova Scotia, Canada. Health promotion international. 2020;35(2):321-30.

15. Weinstein LC, Chilton M, Turchi R, Klassen AC, LaNoue M, Silvero A, et al. 'It's common sense that an individual must eat': Advocating for food justice with people with psychiatric disabilities through photovoice. Health Expect. 2021;24 Suppl 1(Suppl 1):161-73.

16. Gravina L, Jauregi A, Estebanez A, Fernández-Aedo I, Guenaga N, Ballesteros-Peña S, et al. Residents' perceptions of their local food environment in socioeconomically diverse neighborhoods: A photovoice study. Appetite. 2020;147:104543.

17. Freedman DA, Clark JK, Lounsbury DW, Boswell L, Burns M, Jackson MB, et al. Food system dynamics structuring nutrition equity in racialized urban neighborhoods. The American journal of clinical nutrition. 2022;115(4):1027-38.

18. Duffy LR. Hidden heroines: lone mothers assessing community health using photovoice. Health Promot Pract. 2010;11(6):788-97.

19. Kramer L, Schwartz P, Cheadle A, Borton JE, Wright M, Chase C, et al. Promoting policy and environmental change using photovoice in the Kaiser Permanente Community Health Initiative. Health Promot Pract. 2010;11(3):332-9.

20. Young L, Wharf Higgins J. Using participatory research to challenge the status quo for women's cardiovascular health. Nurs Inq. 2010;17(4):346-58.

21. Collie-Akers VL, Fawcett SB, Schultz JA. Measuring progress of collaborative action in a community health effort. Rev Panam Salud Publica. 2013;34(6):422-8.

22. Fawcett SB, Collie-Akers V, Schultz JA, Cupertino P. Community-based participatory research within the Latino health for all coalition. J Prev Interv Community. 2013;41(3):142-54.

23. Madrigal DS, Salvatore A, Casillas G, Casillas C, Vera I, Eskenazi B, et al. Health in my community: conducting and evaluating PhotoVoice as a tool to promote environmental health and leadership among Latino/a youth. Prog Community Health Partnersh. 2014;8(3):317-29.

24. Street J, Cox H, Lopes E, Motlik J, Hanson L. Supporting youth wellbeing with a focus on eating well and being active: views from an Aboriginal community deliberative forum. Australian and New Zealand journal of public health. 2018;42(2):127-32.

25. Kelly RP, Burke J, Waddell S, Lachance L. Increasing Opportunities for Health in a Southeast Michigan Community Through Local Policy Change. Health Promot Pract. 2019;20(1):116-27.

26. Agner J, Pirkle CM, Irvin L, Maddock JE, Buchthal OV, Yamauchi J, et al. The Healthy Hawai'i Initiative: insights from two decades of building a culture of health in a multicultural state. BMC public health. 2020;20(1):141.

27. Walker SC, White J, Rodriguez V, Turk E, Gubner N, Ngo S, et al. Cocreating evidence-informed health equity policy with community. Health services research. 2022;57(S1):137-48.

28. Findholt NE, Michael YL, Davis MM. Photovoice engages rural youth in childhood obesity prevention. Public Health Nurs. 2011;28(2):186-92.

29. Kramer L, Schwartz P, Cheadle A, Rauzon S. Using photovoice as a participatory evaluation tool in Kaiser Permanente's Community Health Initiative. Health Promot Pract. 2013;14(5):686-94.

30. Hackett M, Gillens-Eromosele C, Dixon J. Examining childhood obesity and the environment of a segregated, lower-income US suburb. 2015. . International Journal of Human Rights in Healthcare. 2015(8-4):247-59.

31. John D, Winfield T, Etuk L, Hystad P, Langellotto G, Manore M, et al. Community-Engaged Attribute Mapping: Exploring Resources and Readiness to Change the Rural Context for Obesity Prevention. Prog Community Health Partnersh. 2017;11(2):183-96.

32. Díez J, Gullón P, Sandín Vázquez M, Álvarez B, Martín MDP, Urtasun M, et al. A Community-Driven Approach to Generate Urban Policy Recommendations for Obesity Prevention. International journal of environmental research and public health. 2018;15(4):635.

33. Campbell C, Cornish F, McLean C. Social capital, participation and the perpetuation of health inequalities: obstacles to African-Caribbean participation in 'partnerships' to improve mental health. Ethn Health. 2004;9(4):313-35.

34. Perez G, Della Valle P, Paraghamian S, Page R, Ochoa J, Palomo F, et al. A Community-Engaged Research Approach to Improve Mental Health Among Latina Immigrants: ALMA Photovoice. Health Promot Pract. 2016;17(3):429-39.

35. Thirlwall C, Whitelaw S. Utilising community engagement approaches to influence public mental health policy in a rural setting. Journal of Public Mental Health. 2019.

36. Forenza B, Lardier DT, Reid RJ, Garcia-Reid P, Bermea A. Exploring community stress and empowerment among stakeholders and youth in an urban community. Journal of Human Behavior in the Social Environment. 2019;29(6):705-21.

37. Gullón P, Díez J, Conde P, Ramos C, Márquez V, Badland H, et al. Using Photovoice to Examine Physical Activity in the Urban Context and Generate Policy Recommendations: The Heart Healthy Hoods Study. International journal of environmental research and public health. 2019;16(5).

38. Arredondo E, Mueller K, Mejia E, Rovira-Oswalder T, Richardson D, Hoos T. Advocating for environmental changes to increase access to parks: engaging promotoras and youth leaders. Health Promot Pract. 2013;14(5):759-66.

39. Mier N, Smith ML, Irizarry D, Carrillo-Zuniga G, Lee C, Trevino L, et al. Bridging research and policy to address childhood obesity among border Hispanics: a pilot study. Am J Prev Med. 2013;44(3 Suppl 3):S208-14.

40. Choy LB, Maddock JE, Brody B, Richards KL, Braun KL. Examining the role of a community coalition in facilitating policy and environmental changes to promote physical activity: the case of Get Fit Kaua'i. Transl Behav Med. 2016;6(4):638-47.

41. Winter SJ, Goldman Rosas L, Padilla Romero P, Sheats JL, Buman MP, Baker C, et al. Using Citizen Scientists to Gather, Analyze, and Disseminate Information About Neighborhood Features That Affect Active Living. J Immigr Minor Health. 2016;18(5):1126-38.

42. Rydenstam T, Fell T, Buli BG, King AC, Bälter K. Using citizen science to understand the prerequisites for physical activity among adolescents in low socioeconomic status neighborhoods - The NESLA study. Health Place. 2020;65:102387.

43. Struthers R, Hodge F, Geishirt-Cantrell B, Casken J. Community Mapping: A Tool in the Fight Against Cigarette Smoking on American Indian Reservations. Policy, Politics, & Nursing Practice. 2003;4:295-302.

44. Mahon S, Taylor-Powell E. Case study of capacity building for smoke-free indoor air in two rural Wisconsin communities. Prev Chronic Dis. 2007;4(4):A104.

45. Tanjasiri SP, Lew R, Mouttapa M, Lipton R, Lew L, Has S, et al. Environmental Influences on Tobacco Use Among Asian American and Pacific Islander Youth. Health Promotion Practice. 2013;14(5_suppl):40S-7S.

46. Petteway RJ, Sheikhattari P, Wagner F. Toward an Intergenerational Model for Tobacco-Focused CBPR: Integrating Youth Perspectives via Photovoice. Health Promot Pract. 2019;20(1):67-77.

47. Kingsbury JH, Hassan A. Community-Led Action to Reduce Menthol Cigarette Use in the African American Community. Health Promot Pract. 2020;21(1_suppl):72s-81s.

48. Herd D. Voices from the field: The social construction of alcohol problems in inner-city communities. 2011;38(1):7-39.

49. Drabble L, Herd D. Strategies employed by inner-city activists to reduce alcohol-related problems and advance social justice. J Ethn Subst Abuse. 2014;13(4):362-84.
